# “IT'S GOING TO BE A NEW NORMAL”: OLDER ADULTS' FUTURE PERCEPTIONS DURING COVID-19

**DOI:** 10.1093/geroni/igac059.896

**Published:** 2022-12-20

**Authors:** Bryce Van Vleet, Heather Fuller, Brittany Hofmann, Andrea Huseth-Zosel

**Affiliations:** North Dakota State University, Fargo, North Dakota, United States; North Dakota State University, Fargo, North Dakota, United States; North Dakota State University, Fargo, North Dakota, United States; North Dakota State University, Fargo, North Dakota, United States

## Abstract

This study explores older adults’ perceptions of the future related to COVID-19. Participants (N=76) of a larger study aged 70-97 were asked four times throughout the first year of the pandemic when they thought life would return to normal. Their open-ended responses were coded, and themes were identified at each timepoint. A resilient future perspective was identified at each timepoint; themes of a negative or unstable view of the future emerged over time. Additionally, responses were quantified into a 5-point scale for perceived timescale of a return to normal (5 = very long time) and attitude towards the future (5 = very positive). Repeated measures ANOVA revealed that perceived time to normal increased between the beginning and six months into the pandemic, then decreased by 12 months. Attitudes towards the future became more positive over time. These findings indicated that older adults were largely resilient, if uncertain, about the future.

